# Novel TRPV4 Pathogenic Variant in Severe Metatropic Skeletal Dysplasia: A Case Report

**DOI:** 10.5704/MOJ.2207.021

**Published:** 2022-07

**Authors:** D James, L Subramanian, A Selina, T Palocaren, V Madhuri

**Affiliations:** 1 Department of Paediatric Orthopaedics, Christian Medical College, Vellore, India; 2 Centre for Stem cell Research, Christian Medical College, Vellore, India

**Keywords:** Metatropic dysplasia, homozygous TRPV4 gene variant, severe autosomal recessive form, severe dwarfism

## Abstract

We report an eight-year-old girl with a novel homozygous TRPV4 gene pathogenic variant c.2355G>T p. (Trp785Cys) with mesomelic shortening, odontoid hypoplasia, multiple joint contractures, thoracolumbar kyphosis, pectus carinatum, halberd pelvis, and dumb-bell shaped long bones. The novel variant caused a severe recessive form of metatropic dysplasia.

## Introduction

TRPV4 gene (OMIM # 605427) mutations are known to cause Metatropic dysplasia (MD). This gene, located on chromosome 12q24.11, mediates calcium influx in response to physical, chemical, and hormonal stimuli. This channel plays a significant role in the early development of bones and cartilage. It also plays a role in osmoregulation, and sensation, in particular the pain sensation^1^.

The severe form of MD with autosomal recessive inheritance must be differentiated from the 'classic surviving' autosomal dominant MD and other dysplasias with dwarfism, as this variant can be associated with severe cardio-respiratory involvement. This differentiation of the two entities is needed to guide medical management and to allow caution against surgical interventions^2^.

Here, we report the case of an eight-year-old with a novel homozygous variant in the TRPV4 gene causing metatropic dysplasia. Written informed consent was obtained from the child's parents for reporting this case.

## Case Report

We present an eight-year-old Indian girl, born to second degree consanguineous parents with frontal bossing, hypertelorism, high arch palate, low set ears and a short-webbed neck, with severe short limb and short trunk dwarfism and multiple joint contractures. She was delivered by normal vaginal delivery and weighed 2kg at birth. Her developmental milestones, except locomotion were normal and independent walking was delayed by 2.5 years. There were no visual or hearing deficits. Her activities of daily living were restricted due to difficulty in walking and squatting, with complete cessation of all vocational and recreational activities. Pedigree analysis showed no affected family members in three generations. No history of respiratory distress or neurological symptoms were noted. As a result of the lower limb deformities, she had stopped walking beyond a few steps.

**Fig 1:**
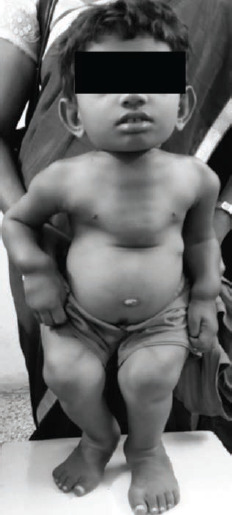
8-years-old girl with severe short trunk dwarfism, multiple joint contractures, pectus carinatum, protuberant abdomen and short flexed lower limbs

She was short statured with her standing and sitting height at 73 and 49cm respectively, with reversed upper to lower segment ratio at 2.04 (Fig. 1). She weighed 14kg, below the 3rd percentile for her age, and her head circumference was 50cm. Short, barrel shaped chest, with thoracolumbar kyphosis and pectus carinatum led her to have severe restriction of chest expansion (1cm). Flexion contractures were noted in all large joints with bilateral elbows, knees, and hip joints with 20°, 60°, and 20° flexion deformities, respectively (Fig. 1). The knee joints were enlarged and bulbous and she had short stubby fingers.

**Fig. 2:**
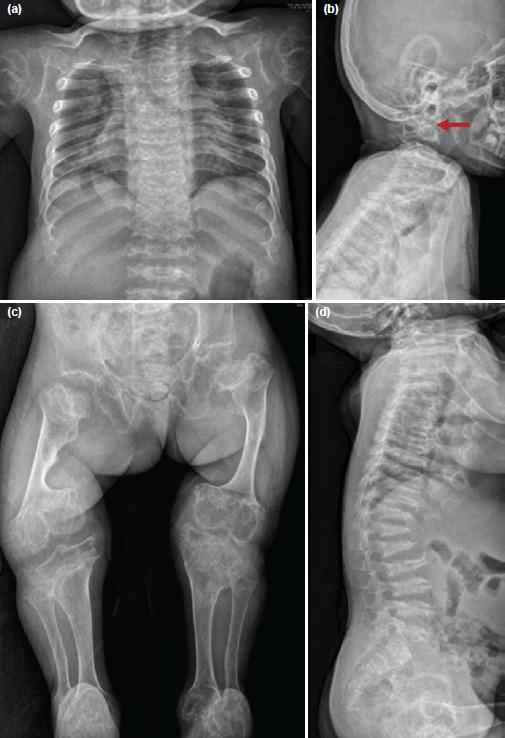
(a) Chest Radiograph shows narrow thorax with broad oar shaped ribs. (b) Lateral cervical spine shows severe odontoid hypoplasia. (c) Halberd Pelvis with short wide ilia, narrow sciatic notches, dysplastic acetabuli, coxa vara and breva, short and thick diaphysis with dumbbell shaped ends, prematurely fused physis, flexion deformity at the knees, fibulae are shorter than tibiae. (d) Lateral spine radiograph shows thoracic lumbar kyphosis, platyspondyly with anterior wedging and characteristic axial split of the lumbar vertebrae body.

Her neck was short and spine radiographs confirmed severe odontoid hypoplasia, upper thoracic lordosis, thoracolumbar kyphosis, and platyspondyly. Chest radiographs revealed a narrow thorax, short and widened ribs. The lumbar vertebral bodies had horizontal splits in the lateral radiographs. All bones showed short and thick diaphysis, prematurely fused physis, dumbbell-shaped long bones, especially femur, valgus deformity of the knees with flexion deformity. Pelvic bones were characterised by wide ilia and narrow sciatic notches, dysplastic acetabuli, and proximal femur with coxa vara and breva (Fig. 2). Echocardiography revealed mild mitral and tricuspid regurgitation.

Based on the short limbs with deformity, spinal involvement and radiology, a clinical diagnosis of metatropic dysplasia, other spondyloepiphyseal dysplasia and Kneist syndrome were considered. The severe flexion deformity of the knees warranted correction with either anterior epiphyseodesis or extension osteotomy of distal femur.

Genomic DNA was isolated from peripheral blood and clinical exome sequencing (CENTOGENE, AG) was conducted on the Illumina platform. A novel homozygous missense variant c.2355G > T in exon 15 of the TRPV4 gene of unknown significance (VUS) was identified. This TRPV4 variant c.2355G > T (p. Trp785Cys) changes the amino acid sequence of the protein from tryptophan to cysteine at position 785 (Table I).

**Table I:** Pathogenicity prediction of the identified mutation (c.2355G>T; p. Trp785Cys) using online in silico tools

In silico tools	Prediction	Score
MutationTaster	Disease-causing	0.99
Polyphen2	Probably damaging	1.000
SIFT	Damaging	0.00
PROVEAN	Deleterious	-11.953
Mutation Assessor	Functional impact-medium	FI score 2.88
Mutpred2	Deleterious	0.798
SNAP2	Effect	70

**Fig. 3:**
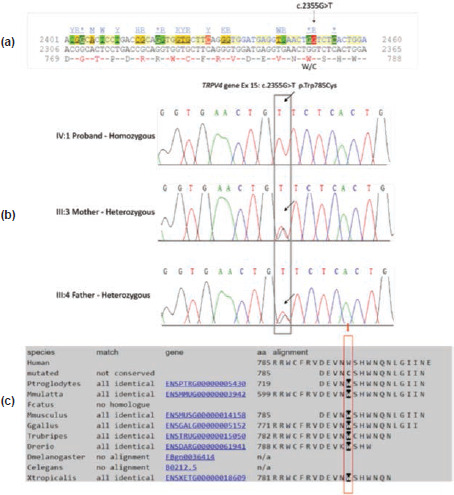
(a) The amino acid sequence of the TRPV4 gene and the position of the novel variant identified c.2355G > T p. (Trp785Cys) is shown. (b) Electropherogram of the TRPV4 gene variant position: Proband showing homozygous c.2355G > T p. (Trp785Cys) missense variant at exon 15 of TRPV4 gene. Parents were heterozygous. (c) Tryptophan W at position 785 of TRPV4 gene is conserved in different species.

Sanger sequencing of the identified variant was done for the proband and the parents to confirm the variant and segregation analysis. The DNA sequence of the TRPV4 gene was obtained from the ENSEMBL database (http://asia.ensembl.org. Primers were designed by Primer, and samples were sequenced using ABI 3130 genetic analyser [Applied Biosystems, California, USA]. The nucleotide sequences were assessed using Mutation Surveyor from Softgenics, and BioEdit software. The cDNA transcript of the TRPV4 gene (Transcript id: ENST00000418703.6) with the novel missense variant c.2355G > T p. (Trp785Cys) was identified (Fig. 3a). Segregation analysis showed heterozygous variants in the parents (Fig. 3b). Fig. 3c shows the corresponding wild-type amino acid residue 785 tryptophan of the TRPV4 gene, which is conserved in different species.

## Discussion

Metatropic dysplasia is caused by deleterious mutations in the TRPV4 gene and is known to be autosomal dominant.

Generally considered an autosomal dominant entity, the recessive variant of severe 'classic surviving' MD has not been reported, though homozygous variants are reported in neuromuscular TRPV4 disorder^3^.

Phenotypically and radiologically, the child had features of severe metatropic dysplasia supported by the novel mutation variant c.2355G>T p. (Trp785Cys) in exon 15 of the TRPV4 gene. Based on clinical and radiological features, other skeletal dysplasias like spondyloepiphyseal dysplasia congenita, Strudwick type, Kneist dysplasia, Morquio's syndrome, achondroplasia, pseudoachondroplasia, and fibrochondrogenesis also qualify as differential diagnoses. A skeletal survey is essential to differentiate severe short limb, short trunk dwarfism of MD from other similar non-lethal skeletal dysplasias with overlapping clinical and radiological features, especially in an older child.

Severe dwarfism, protuberant abdomen, oar-shaped ribs, and platyspondyly of morquio syndrome can be differentiated from MD by its characteristic central vertebral beaking, preserved intervertebral space, pointy metacarpals, delayed epiphyseal ossification, and elevated urinary glycosaminoglycans.

Lack of characteristic dysmorphic facial features, trident hand, preserved vertebral height with a progressive decrease in the interpedicular distance differentiates the MD phenotype from achondroplasia. Multiple joint hyperlaxity seen in pseudoachondroplasia is contrasting to the multiple joint deformities seen in this patient.

The six different skeletal manifestations of TRPV4 variants progressing from mild to severe types are familial digital arthropathy, brachyolmia, spondylometaphyseal dysplasia Kozlowski type, and Maroteaux type (pseudo-Morquio syndrome type 2), metatophic dysplasia, and parastremmatic dysplasia. The features that differentiate MD from the above diagnoses are the absence of dumbbell-shaped femur in spondylometaphyseal dysplasia of Kozlowski type and Maroteaux type and bowing of long bones with severely deformed metaphyses and radiolucent epiphysis in parastremmatic dysplasia^4,5^.

Patients with the 'classical surviving' autosomal dominant variety of MD are likely to achieve an average adult height ranging from 110 to 145cms. However, premature growth arrest and plateauing of growth as early as six to eight years have been described^5^. Surgical interventions become necessary to address severe functional impairment arising from secondary to progressive kyphoscoliosis and early onset of joint stiffness^4^. Given the predilection for respiratory arrest, even without any underlying predisposing factor in the autosomal recessive form, this novel late-presenting recessive variant case gains significant importance. A high degree of caution must be exercised in determining surgical intervention in this variant as it is likely to have similar respiratory complications as in the severe infantile lethal recessive form.

In conclusion, MD is a form of autosomal dominant skeletal dysplasia with TRPV4 mutation, and we report a recessive inheritance of novel form TRPV4 mutation. The striking radiological features distinguishes it from other short-statured skeletal dysplasias. The severely restricted lung capacity and cervical spine instability cause significant morbidity and mortality risk.
